# Intact fetal ovarian cord formation promotes mouse oocyte survival and development

**DOI:** 10.1186/1471-213X-10-2

**Published:** 2010-01-08

**Authors:** Cory R Nicholas, Kelly M Haston, Renee A Reijo Pera

**Affiliations:** 1 Institute for Stem Cell Biology and Regenerative Medicine, Department of Obstetrics and Gynecology, Stanford University, Palo Alto, CA, USA

## Abstract

**Background:**

Female reproductive potential, or the ability to propagate life, is limited in mammals with the majority of oocytes lost before birth. In mice, surviving perinatal oocytes are enclosed in ovarian follicles for subsequent oocyte development and function in the adult. Before birth, fetal germ cells of both sexes develop in clusters, or germline cysts, in the undifferentiated gonad. Upon sex determination of the fetal gonad, germ cell cysts become organized into testicular or ovarian cord-like structures and begin to interact with gonadal somatic cells. Although germline cysts and testicular cords are required for spermatogenesis, the role of cyst and ovarian cord formation in mammalian oocyte development and female fertility has not been determined.

**Results:**

Here, we examine whether intact fetal ovarian germ and somatic cell cord structures are required for oocyte development using mouse gonad re-aggregation and transplantation to disrupt gonadal organization. We observed that germ cells from disrupted female gonad prior to embryonic day e13.5 completed prophase I of meiosis but did not survive following transplantation. Furthermore, re-aggregated ovaries from e13.5 to e15.5 developed with a reduced number of oocytes. Oocyte loss occurred before follicle formation and was associated with an absence of ovarian cord structure and ovary disorganization. However, disrupted ovaries from e16.5 or later were resistant to the re-aggregation impairment and supported robust oocyte survival and development in follicles.

**Conclusions:**

Thus, we demonstrate a critical window of oocyte development from e13.5 to e16.5 in the intact fetal mouse ovary, corresponding to the establishment of ovarian cord structure, which promotes oocyte interaction with neighboring ovarian somatic granulosa cells before birth and imparts oocytes with competence to survive and develop in follicles. Because germline cyst and ovarian cord structures are conserved in the human fetal ovary, the identification of genetic components and molecular mechanisms of pre-follicle stage germ and somatic cell structures may be important for understanding human female infertility. In addition, this work provides a foundation for development of a robust fetal ovarian niche and transplantation based system to direct stem cell-derived oocyte differentiation as a potential therapeutic strategy for the treatment of infertility.

## Background

Ovarian follicles, consisting of an oocyte and surrounding somatic granulosa cells, are essential for oocyte survival and maturation [1,2]; however, the role of pre-follicle stage fetal ovarian germ and somatic cell structures in directing mammalian oocyte development has not been well defined. Prior to follicle formation, germ cell precursors of several species have been observed to develop in cysts [3,4]. Germline cyst formation occurs by incomplete cytokinesis during mitosis, resulting in the connection of daughter cells by intercellular bridges. In *Drosophila*, cyst formation and intercellular bridge-mediated transport of organelles and RNA determine oocyte fate and fertility of the adult female [5-7].

Mouse primordial germ cells (PGCs) also develop in cyst-like clusters following colonization of the genital ridge and subsequent cell division. The maximum number of germ cell clusters is detected on embryonic day e13.5 just prior to commitment, or maturation, of PGCs to an oocyte developmental program and successive entry into meiosis [8]. Upon commitment, oocyte clusters become organized into poorly defined ovarian, or ovigerous, cord-like structures containing oocytes and pre-granulosa somatic cells [1]. Shortly after birth, ovarian cords and cysts break down into follicles characterized by oocyte apoptosis, the organization of granulosa cells around surviving oocytes, and basement membrane remodeling [9-11]. Follicle formation therefore appears to require intricate synchronization of oocyte precursor germ cells and pre-granulosa somatic cells [12,13].

Although ovarian cyst and cord break down has been implicated in follicle formation [10], it is not yet known whether cyst and/or cord formation is required for mammalian oocyte development before follicle formation. Previously, intercellular bridges were found to be dispensable for female fertility in mammals [14]; however, germ cell cyst formation was not obstructed in mice lacking bridges, and, therefore, the requirement of cyst and cord formation for oocyte development could not be determined. Here, we use fetal ovary re-aggregation and transplantation to directly perturb ovarian cysts and cords, and we demonstrate that intact ovarian cord formation and maturation promote oocyte survival and development.

## Results

### Intact ovary maturation promotes oocyte development

To investigate the role of intact ovarian cords in promoting oocyte development, we dissected fetal to newborn stage mouse female genital ridges and ovaries, dissociated the tissues to single cells, re-aggregated the dissociated cells, and then transplanted the re-aggregated tissues under the kidney capsules of bilaterally ovariectomized immuno-deficient mouse recipients for three weeks. Post-transplantation, we constructed a timeline denoting the competence of gonads at different stages of development to support oocyte survival and maturation. For this purpose, transplanted grafts from e11.5 to post-natal P2 female gonadal stages were sectioned and analyzed for the presence of oocyte-containing follicles by hematoxylin and eosin staining following re-aggregation and transplantation as described [15]. Follicles containing oocytes with visible nuclei were counted on every 10^th ^section, and the average follicle number per graft (n = 3 grafts per gonadal stage) was normalized either per section or per weight (mg) of graft tissue. No significant difference was found between normalization methods (per weight normalization not shown).

**Figure 1:**
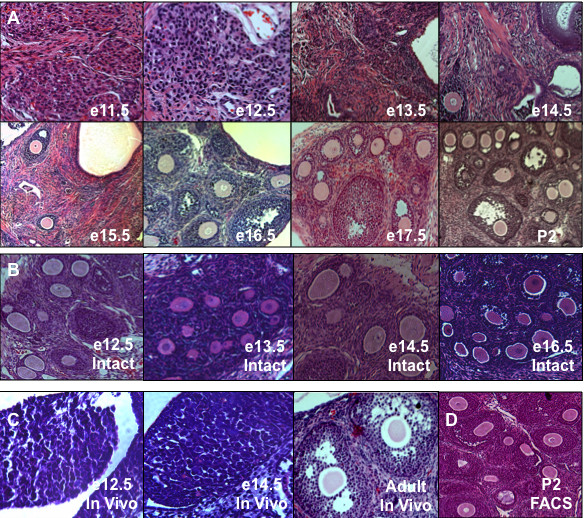
**Female gonadal re-aggregation prior to ovary maturation impaired oocyte development**. (A) Female mouse genital ridges and ovaries from e11.5 (embryonic day) to P2 (post-natal day) were disrupted by re-aggregation, transplanted under the kidney capsule for three weeks, and sections were examined by H&E stain for oocyte development in follicles in comparison to intact (B) gonadal transplant controls. (C) For comparison, *in vivo *control endogenous time points are shown to illustrate e12.5 genital ridge, pre-follicle formation e14.5 differentiating ovary, and post-follicle formation adult ovary. (D) P2 ovaries were also FACS sorted prior to re-aggregation to ensure a single cell suspension and an equivalent dissociation method. 100× magnification.

**Figure 2:**
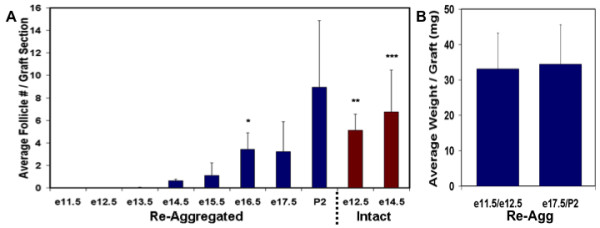
**Quantification of re-aggregation induced impairment of oocyte development**. (A) The average number of oocytes in follicles per graft, normalized to the number of sections counted, was determined following three weeks transplantation of intact or re-aggregated samples. No oocytes were detected in re-aggregated samples prior to e13.5. Oocyte numbers increased in re-aggregated samples after e13.5 in parallel to the increasing age of ovary donors, however, oocyte numbers were still significantly reduced from e13.5 to e15.5 compared to intact control samples. (B) Average weight per graft following transplantation of re-aggregated early and late gonadal stages demonstrating equivalent weights. Error bars represent s.d. (n = 3, 150 sections/graft). * = p < 0.05 between e16.5 and e15.5 re-aggregated. **/*** = p < 0.05 between e12.5/e14.5 intact and re-aggregated, respectively.

We observed that the number of oocytes in follicles detected following re-aggregation and transplantation was not equal and generally increased with the age of embryos used as ovary donors (Figures 1A and 2A). Oocytes that were not enclosed in follicles were never detected by VASA immunostaining analysis of any ovarian stage following three weeks of transplantation (data not shown). In contrast to significant folliculogenesis following transplantation of intact gonadal controls (Figure 1B) or endogenous adult ovary *in vivo *controls (Figure 1C), ovarian follicles were not detected from re-aggregated e11.5 or e12.5 female genital ridges (Figures 1A and 2A). Rare oocytes in follicles were observed beginning from re-aggregated e13.5 ovaries, and follicle number increased from e13.5 to e16.5, with a significant elevation from e15.5 to e16.5 (Figure 2A). Robust folliculogenesis was supported by re-aggregated e16.5 to P2 ovarian stages.

E11.5 to e14.5 transplants were then sectioned through and examined in their entirety to ensure that the absence of follicles observed before e13.5 could not be due to dilution of oocytes by ovarian somatic cells that had greater proliferative potential at these earlier gonadal stages. We confirmed an absence of follicles from e11.5 and e12.5 transplants and detected similarly low numbers of oocytes from e13.5 and e14.5 samples (data not shown). In fact, the average weight of grafts was nearly identical between e11-12.5 and e17.5-P2 gonadal stage transplants (Figure 2B). Furthermore, the re-aggregation impairment of oocyte development from early ovarian stages was not a non-specific effect of physical gonadal disruption but rather a biological function of gonadal stage prior to the disruption, as oocyte development occurred following re-aggregation and transplantation of later stage ovaries that were treated identically. To rule out the possibility of later stage ovary resistance to enzymatic dissociation to single cells as being responsible for robust oocyte development, newborn ovaries were dissociated as described, then filtered through a 40 μm membrane to remove cell clumps, and FACS sorted for single cells based on forward and side scatter parameters to exclude doublets and to ensure a single cell suspension before re-aggregation. Indeed, FACS sorted and re-aggregated newborn ovaries still supported robust oocyte development of equivalent numbers of follicles after transplantation (36 follicles/section for P2 and P2 FACS samples) (Figure 1D). Thus, the results of the re-aggregation time course indicated that pre-e13.5 stage gonadal cells were not yet competent to support oocyte development following disruption. We concluded that intact ovarian development on e13.5 was important for oocyte survival and maturation in follicles.

### Intact ovaries are not required for oocyte meiotic entry or progression

Re-aggregated female germ cells became competent to develop as oocytes in follicles after e13.5 and corresponded to the time of female sex determination and PGC commitment to an oocyte developmental program on e13.5 *in vivo *[16]. Soon after their commitment, oocytes begin to enter meiotic prophase I from e13.5 to e16.5 [17,18], representing an early hallmark of fetal oocyte development and also paralleling the increase in oocyte development observed in our re-aggregation timeline.

**Figure 3:**
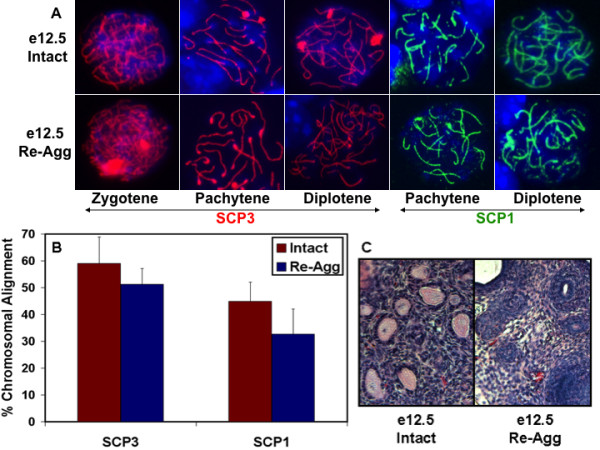
**Genital ridge re-aggregation did not affect oocyte meiotic entry or progression but disrupted oocyte survival and development in follicles**. (A) Intact or re-aggregated (re-agg) e12.5 female genital ridges were transplanted for three days, and germ cells were subsequently analyzed for entry and progression through stages of meiosis prophase I by SYCP3 and SYCP1 immunostaining of chromosome synapsis. Blue is DAPI. 630× magnification. (B) Intact or re-aggregated e12.5 samples on day three of transplantation were quantified for the percentage of cells in meiotic prophase I as determined by SYCP chromosomal alignment localization by immunofluorescence. Error bars represent s.d. (n = 3, 100 cells/graft). No statistically significant difference was detected. (C) By 12 days of transplantation, many primordial and primary follicles were observed from the intact e12.5 genital ridge but none were detected from the re-aggregated genital ridge by H&E stain. 200× magnification.

Consequently, we examined pre-e13.5 stage germ cells for meiotic entry and progression as a measure of oocyte commitment, and we observed that re-aggregated germ cells were still competent to commit to an oocyte developmental program and enter meiosis following transplantation (Figure 3A). Re-aggregated or intact e12.5 female genital ridges transplanted for three days and assayed by immunofluorescence for expression and localization of synaptonemal complex protein markers of meiotic chromosome synapsis displayed an almost equivalent percentage of meiotic cells. Both samples contained oocytes that expressed markers of zygotene to diplotene stages of meiotic prophase such as chromosomal alignment of SYCP3 (SYnaptonemal Complex Protein 3; 59% in intact and 51% in re-aggregated), and markers of pachytene to diplotene stages such as chromosomal localization of SYCP1 (45% in intact and 33% in re-aggregated) (Figure 3B). Thus, the complete absence of oocytes and follicles from re-aggregated e12.5 samples was not due to lack of oocyte commitment and entry into meiosis.

To determine the extent of meiotic progression, we examined e12.5 genital ridges after five days of transplantation and detected only a small percentage of cells that stained positive for chromosomal elongated localization of SYCP3 and SYCP1 for either re-aggregated or intact control samples (6% in intact and 14% in re-aggregated for SYCP3; 4% in intact and 2% in re-aggregated for SYCP1) (data not shown). These results indicated that germ cells from both intact and re-aggregated e12.5 samples entered and progressed through meiotic prophase I to dictyate arrest as oocytes and subsequently down-regulated the expression of SYCP proteins. Down-regulation of SYCP1 upon successful meiotic progression of oocytes has been suggested to facilitate follicle formation [19]. Therefore, PGC commitment to oogenesis and oocyte progression through meiotic prophase I did not appear to be affected by re-aggregation, although we cannot eliminate the possibility of defects in DNA mismatch or double strand break repair. In addition, neither complete sex reversal to spermatogenesis and testicular structures nor ectopic PGC reprogramming and teratoma formation following genital ridge disruption were ever observed.

### Intact ovary maturation supports oocyte survival before follicle formation

Although germ cells entered and progressed through early stages of meiosis as committed oocytes in the three to five day transplants from re-aggregated e12.5 female genital ridge, the oocytes did not survive at three weeks of transplantation. We hypothesized that re-aggregation either directly impaired oocyte survival or impaired the development of ovarian somatic cells and indirectly led to oocyte degeneration subsequent to an obstruction in the formation of follicles. Thus, intact and re-aggregated e12.5 genital ridges were examined along a time course of transplantation to determine when oocyte loss occurred with respect to follicle formation and ovarian somatic granulosa cell development, and to elucidate a germ cell or somatic cell basis for the re-aggregation impairment of oocyte survival.

**Figure 4:**
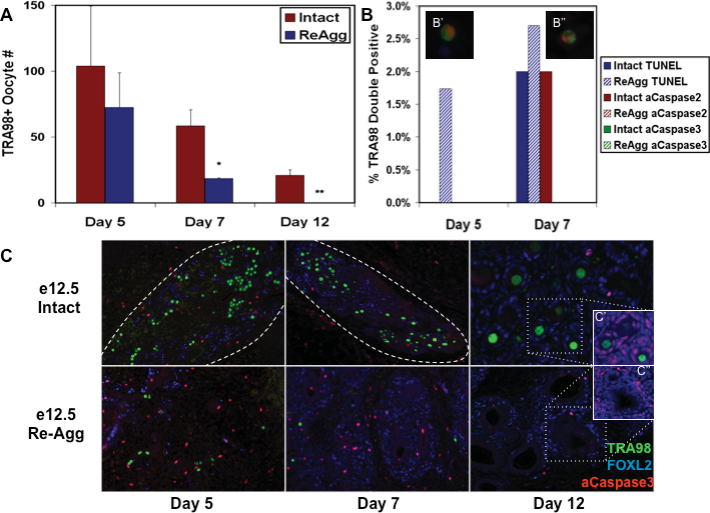
**Re-aggregation obstructed intact ovarian cord structure and induced Caspase-independent oocyte loss before follicle formation**. (A) The numbers of surviving oocytes per graft were determined by TRA98 immunofluorescence following five, seven, and 12 days of intact or re-aggregated (re-agg) e12.5 female genital ridge transplantation revealing oocyte loss by d7 before follicle formation on d12. Error bars represent s.d. (n = 3) */** = p < 0.05 between d7/d12 re-aggregated and intact, respectively. (B) Re-aggregation induced oocyte loss was Caspase-independent. The percentage of TRA98+ oocytes (100 cells/graft) undergoing apoptosis was assayed by TUNEL and active Caspase2 or Caspase3 co-immunofluorescence following five to seven days of transplantation. Intact = solid bars. Re-aggregated = striped bars. TUNEL+ (red) apoptotic oocytes co-stained for TRA98 (green) were detected in re-aggregated samples following 5 (B') and 7 (B'') days of transplantation (C) Ovarian cord structure was examined by TRA98 germ cell and FOXL2 granulosa somatic cell immunofluorescence over 12 days of e12.5 female genital ridge transplantation. Oocytes and granulosa cells clustered together in defined ovarian cord-like structures (dashed lines) following five to seven days of intact sample transplantation, and ovarian follicles were detected by day twelve. Re-aggregated samples did not form ovarian cords or follicles during the time course, and TRA98+ oocytes were not detected after day seven in re-aggregated transplants. Active Caspase3 was not detected in oocytes. (C' and C'') Clusters containing FOXL2+ cells were observed following re-aggregation, however, FOXL2+ cells were only detected at the perimeter of these clusters, and neither FOXL2+ nor TRA98+ cells were ever observed at the interior in contrast to intact ovarian follicles. C' and C'' panels: DAPI - blue, FOXL2 - red, and TRA98 - green. 100× magnification.

Endogenous ovarian follicle formation occurs just after birth in mice. As expected, follicles were not detected from either intact or re-aggregated e12.5 samples after only five to seven days of transplantation, comparable to e17.5 to e19.5 pre-natal stages; however, many primordial follicles were identified from the intact e12.5 female genital ridge beginning on day 12 of transplantation. In contrast, ovarian follicles were not observed from the re-aggregated e12.5 genital ridge on day 12 (Figure 3C). To further characterize oocyte loss, we investigated the decline in oocyte number by TRA98 oocyte marker immunostaining of intact and re-aggregated e12.5 female genital ridges after transplantation for five, seven, and 12 days. Intact control transplants exhibited gradual oocyte loss during the time course, similar to oocyte loss *in vivo*, with 20% of oocytes from day five of transplantation surviving to day 12 and forming follicles. Previously, endogenous peri-natal oocyte loss *in vivo *was reported to occur through programmed ovarian germ cell cyst break down, with 33% of oocytes surviving to form primordial follicles [10,20]. In contrast, re-aggregation resulted in accelerated oocyte loss and significant reduction in oocyte number by day seven of transplantation with no oocytes surviving to day 12 (Figure 4A). Thus, oocytes from re-aggregated genital ridge were being lost by day seven of transplantation before follicle formation on day 12 and suggested an oocyte-specific impairment as a cause rather than a consequence of folliculogenesis obstruction. Furthermore, FOXL2+ ovarian somatic granulosa cells survived and were detected at all time points of re-aggregated transplantation in similar numbers to intact samples despite the absence of TRA98+ oocytes, in line with prior observations [21,22], confirming a re-aggregation induced oocyte-specific deficiency (Figure 4C).

To determine the mechanism of oocyte loss after re-aggregation, we examined markers of apoptosis following transplantation of intact or re-aggregated e12.5 female genital ridge and detected evidence of Caspase-independent apoptosis. Although activated Caspase2 and 3 apoptotic pathways have been previously implicated in oocyte death [23-29], TRA98+ oocytes expressing active Caspase2 or Caspase3 were not detected following re-aggregation. However, 2% of oocytes expressed active Caspase2 in intact genital ridge, and active Caspase3 was observed in some FOXL2+ granulosa cells from both samples (Figure 4B, C). In contrast, we detected no TRA98/TUNEL double positive apoptotic oocytes in the intact samples after five days of transplantation, while 1.7% of the TRA98+ oocytes were TUNEL+ in the re-aggregated genital ridge (Figure 4B, B'). On day seven, we detected 2% and 2.7% TUNEL+ apoptotic oocytes in intact and re-aggregated samples, respectively (Figure 4B, B''). Therefore, a small increase in TUNEL+ apoptotic oocytes was observed following re-aggregation indicating that TUNEL+, Caspase-independent apoptosis may play a role in re-aggregation induced oocyte loss. However, other mechanisms of cell death or loss, such as necrosis and/or autophagy, are likely to be involved in oocyte reduction/depletion as well. Additionally, oocyte differentiation can not be excluded.

### Intact ovarian cord formation promotes oocyte survival and development

**Figure 5:**
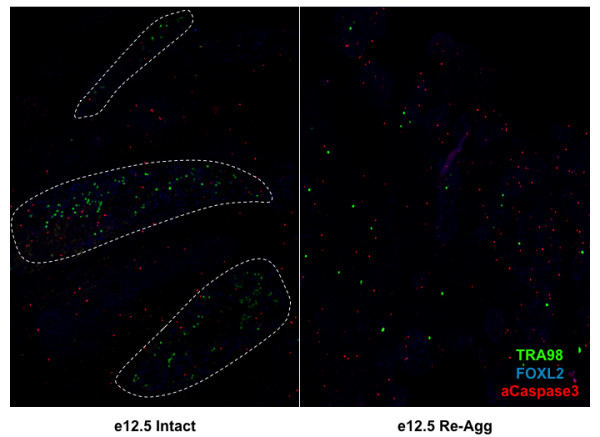
**Re-aggregation impaired intact ovarian cord formation**. Low magnification (40×) analysis of ovary structure by TRA98 germ cell and FOXL2 granulosa somatic cell immunofluorescence after five days of e12.5 female genital ridge transplantation revealed the striking difference in ovary organization between intact and re-aggregated samples. Intact sample oocytes and granulosa cells clustered together in defined ovarian cord-like structures (dashed lines), in contrast to re-aggregated samples which did not form ovarian cords containing oocyte clusters. Active Caspase3 was not detected in oocytes.

Having demonstrated that oocytes are directly impaired and lost before follicle formation following disruption of the e12.5 female genital ridge, we next explored the properties of the ovary that develop on e13.5 and enable oocyte survival after re-aggregation and transplantation. We observed that re-aggregation resulted in the disorganization of ovary structure and corresponded to the absence of ovarian cord formation and subsequent oocyte loss. In transplants of intact e12.5 female genital ridge, TRA98+ oocytes and FOXL2+ granulosa cells co-localized to large clusters or ovarian cords by five days of transplantation that correlated with successive oocyte survival and follicle formation on day 12. However, ovarian cords were significantly disrupted in transplants from re-aggregated e12.5 female genital ridge, as clusters of TRA98+ oocytes and FOXL2+ granulosa cells were not observed at any time point after transplantation (Figure 4C). Instead, individual TRA98+ oocytes were distributed evenly throughout the re-aggregated gonad after five to seven days of transplantation and were not detected by day 12 (Figures 4C and 5).

In contrast, FOXL2+ granulosa cells were found to re-organize in clusters following re-aggregation, however, these granulosa cell domains were not specifically enriched for oocytes and exhibited a distinctly different morphology from intact ovarian follicles. In contrast to FOXL2+ granulosa cells throughout the somatic cell layers of intact ovarian follicles and adjacent to the oocyte, FOXL2+ cells were confined to the perimeter of these re-aggregated clusters with several layers of FOXL2-, DAPI+ cells at the interior facing an oocyte-less lumen (Figure 4C', C''). Oocytes were occasionally observed in a FOXL2+ cluster, but this non-specific interaction was the result of random oocyte distribution and dense granulosa cell domains after disruption and re-aggregation (Figure 5). Thus, the survival and re-organization of granulosa cells, and oocyte lack thereof, was indicative of an oocyte-specific deficiency induced by re-aggregation that corresponded to disruption of ovarian cord formation.

**Figure 6:**
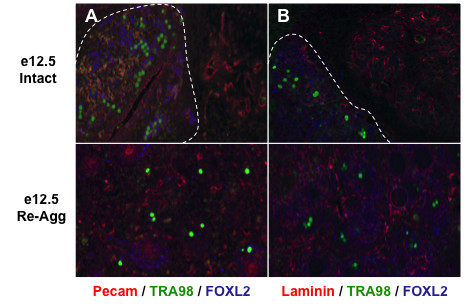
**Oocyte loss was associated with ovary disorganization following re-aggregation**. (A, B) Immunofluorescence revealed TRA98+ oocytes and FOXL2+ granulosa cells in ovarian cord-like structures (dashed lines) primarily sequestered from PECAM+ endothelial cells (A) and Laminin+ basment membrane (B) following five days of intact e12.5 female genital ridge transplantation. Along with disrupted ovarian cord formation, re-aggregation also resulted in a uniform distribution of PECAM+ and Laminin+ cells amongst dispersed oocytes and granulosa cells that correlated with subsequent oocyte loss. 100× magnification.

Re-aggregation also resulted in ovary disorganization of other somatic cell types that correlated with ovarian cord disruption and oocyte loss. In intact e12.5 female genital ridge transplants, somatic cells expressing PECAM (Figure 6A) and Laminin (Figure 6B), markers of endothelial cells and basement membrane, respectively, were predominantly separated from oocytes by intact ovarian cords. Of note, we did not detect PECAM expression in oocytes following five days of e12.5 genital ridge transplantation (equivalent to e17.5), confirming a prior report that PECAM expression is extinguished in oocytes by e16 and, thus, can be used as a somatic cell-specific marker at this stage [30]. In re-aggregated samples, in contrast, somatic cells expressing PECAM and Laminin were instead uniformly interspersed, along with randomly dispersed oocytes and granulosa cell clusters, by day five of transplantation (Figure 6A, B). Therefore, in addition to disruption of ovarian cords, disorganized somatic cell contact- and/or paracrine signaling-mediated oocyte toxicity induced by the re-aggregation could also play a role in the lack of oocyte survival.

To distinguish ovarian cord disruption from somatic cell toxicity mechanisms of re-aggregation-induced oocyte loss, e12.5 female genital ridges were again dissociated to obstruct ovarian cord formation. Instead of re-aggregation, germ cells were purified from e12.5 somatic cells, which may be inhibitory, and were then co-aggregated with later stage e16.5 and P2 ovaries. Notably, we previously found disrupted and re-aggregated e16.5 and P2 ovarian stages capable of supporting robust oocyte survival and development as characterized by ovarian somatic cell organization and folliculogenesis (Figures 1A and 2A). Therefore, the inability of e12.5 germ cells to survive as oocytes and contribute to follicle formation and development with later stage ovarian somatic cells would reflect an oocyte-specific deficiency. As such, we used a transgenic *Oct4*-GFP mouse model [31] to purify e12.5 germ cells from the female genital ridge by fluorescence activated cell sorting (FACS). Purified GFP+ e12.5 germ cells were then directly co-aggregated with e16.5 and P2 ovarian tissues and transplanted for three weeks. Following transplantation, however, we could not identify any GFP+ germ cell-derived oocytes (data not shown) indicating a lack of germ cell-specific competency at the e12.5 stage to support oocyte survival and development.

**Figure 7:**
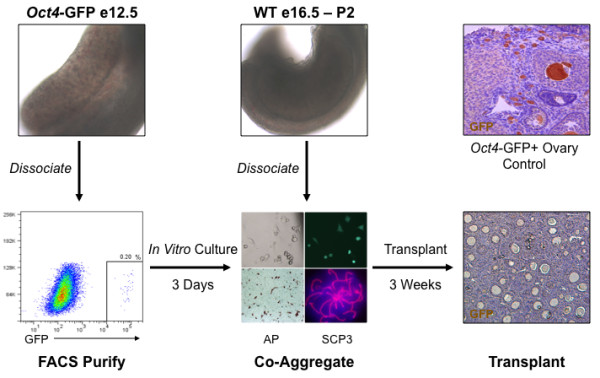
**Disruption of intact ovarian structure by re-aggregation resulted in oocyte loss due to an oocyte-specific deficiency**. To distinguish between oocyte-specific or somatic cell-specific mechanisms of re-aggregation-induced oocyte loss, germ cells from e12.5 female genital ridges of *Oct4*-GFP transgenic mice were purified from somatic cells by FACS, and GFP+ and alkaline phosphatase (AP+) germ cells were cultured *in vitro *for three days to induce meiosis (SYCP3+) and to synchronize germ cells with later ovarian stages. Subsequently, cultured GFP+ germ cells were co-aggregated with permissive e16.5 or P2 stage ovarian tissue and transplanted for three weeks. In contrast to *Oct4*-GFP transgenic ovary controls with robust oocyte-specific GFP expression, e12.5-derived GFP+ oocytes were not detected after transplantation and indicated a re-aggregation induced oocyte-specific impairment following pre-ovarian genital ridge disruption.

Although our results advocated an oocyte-specific impairment, we next sought to rule out e12.5-derived oocyte loss due to unsynchronized germ and somatic cell stages. Previous studies suggested that pre-natal oocyte development requires synchronization of germ and somatic cells [12,13], in contrast to post-natal stages where synchronization is not required [32]. Accordingly, e12.5 *Oct4*-GFP+ germ cells were isolated by FACS and synchronized with later stage ovarian somatic cells by *in vitro *culture for three days to induce oocyte entry into and progression through meiosis that corresponded to an e15-17.5 stage of ovary development. GFP+ germ cells were cultured on cell culture inserts in media supplemented with several factors adapted from a prior report of *in vitro *PGC survival and entry into meiosis in the absence of somatic cells [33]. Purified germ cells were alkaline phosphatase positive and continued to express GFP *in vitro*, confirming their germ cell identity (Figure 7). Furthermore, 78% of germ cells had entered meiosis by day three of culture as determined by SYCP3 and SYCP1 expression and elongated chromosomal localization, indicating a zygotene to pachytene stage of meiosis prophase I and confirming oocyte maturation *in vitro*. Meiotic oocytes were subsequently co-aggregated with permissive e16.5 or P2 ovaries and transplanted. However, we were still unable to detect e12.5 germ cell-derived GFP+ oocyte survival and development even though many GFP- oocytes from e16.5 and P2 ovaries were observed in follicles (Figure 7). Thus, we confirmed a re-aggregation-induced, e12.5-derived deficiency that was oocyte-intrinsic and corresponded to a disruption of intact ovarian cord formation, demonstrating a critical window from e13.5 to e16.5 of oocyte development in the intact ovary for subsequent oocyte survival and maturation in follicles.

Furthermore, preliminary results have been obtained to further confirm an oocyte-specific impairment by performing a reverse experiment. Ovarian tissue from permissive e16.5 embryos was co-aggregated with somatic mesonephros tissue from non-permissive e13.5 ovaries and transplanted for 12 days. Oocytes from e16.5 ovaries have already been exposed to intact ovarian cord structures beginning at e13.5 of development. Based on our previous results suggesting oocyte survival following re-aggregation to be an oocyte-intrinsic function of intact ovarian cord development from e13.5 to e16.5, we expected that these e16.5 oocytes would be competent to survive and form follicles, even when in the presence of somatic tissue from the genital ridges of pre-competent stages. Indeed, e16.5 oocytes survived to form follicles and further suggested an oocyte-intrinsic impairment following the disruption of intact ovarian cord structure during early genital ridge and ovarian stages (data not shown). Nevertheless, we do not exclude the possibility that re-aggregated somatic cells from the early female genital ridge and ovary contribute to the inhibition of oocyte survival and development along with the absence of intact ovarian cords.

## Discussion

In the present study, we demonstrate that intact ovarian cord formation promotes oocyte development. The female embryonic genital ridge was not competent to support oocyte survival when disrupted prior to e13.5 ovary maturation and cord formation. Furthermore, oocyte competence to survive and develop in follicles following ovary re-aggregation paralleled the duration of oocyte development within intact fetal ovarian cords from e13.5 to e16.5 prior to their disruption. Pre-ovarian genital ridge re-aggregation did not affect germ cell sex determination or commitment to oogenesis and entry into meiosis (Table 1). However, pre-ovarian e12.5 re-aggregation triggered oocyte loss before follicle formation, which was independent of Caspase pathway activation, and was correlated with obstruction of ovarian cord formation and ovary disorganization. In contrast, granulosa cells survived re-aggregation and exhibited re-organization, indicating an oocyte-specific deficiency. Moreover, heterochronic experiments of pre-ovarian stage germ cell co-aggregation with later stage ovary somatic cells, that were competent to direct follicle formation following re-aggregation, did not support e12.5-derived oocyte survival and confirmed a critical role for e13.5-e16.5 intact ovarian structures in programming oocytes with intrinsic competence to survive and develop in follicles.

**Table 1:** Summary of analysis and results following e12.5 female genital ridge transplantation.

Days of Transplantation	**Analysis**^**a**^	Re-Agg Gonad	Intact Gonad	In Vivo^**b **^Control
**3**	Meiosis	Zygotene-pachytene of meiosis I	Zygotene-pachytene of meiosis I	Zygotene-pachytene of meiosis I [18]

**5**	Ovarian Cord Formation	Ovarian cords do not form and ovary disorganized	Ovarian cords form with distinct organization of oocyte and granulosa cell clusters	Ovarian cords form with distinct organization of oocyte and granulosa cell clusters [1]

**7**	Oocyte Survival	Oocyte numbers significantly reduced compared to intact gonad	Oocyte numbers reduced compared to day 5	Oocyte numbers decline due to programmed breakdown [10]

**12-21**	Oocyte Development in Follicles	Oocytes do not survive to day 12 and follicles not observed	Oocytes survive, form follicles, and mature	Oocytes survive, form follicles, and mature [2]

a For all experiments, n = 3 transplants with 4 embryos or 8 gonads per transplant

b In vivo control refers to e15.5, e17.5, e19.5, and P4 - P14 stages

**Figure 8:**
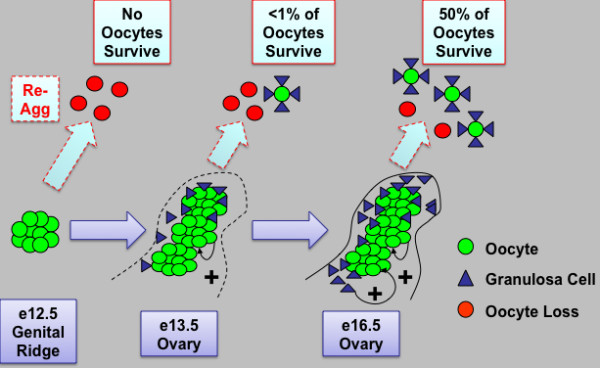
**Model of intact ovarian cord formation and maturation promoting oocyte survival and development in follicles**. Prior to ovary development on e13.5, germ cells located in cysts in the e12.5 female genital ridge are not yet competent to survive and form follicles when re-aggregated (re-agg). However, ovary maturation on e13.5 and development of intact ovarian cord structures containing oocyte and somatic granulosa cell clusters are sufficient to permit some oocyte survival and follicle formation upon re-aggregation. By e16.5, intact ovarian cord-enclosed meiotic oocyte clusters and granulosa somatic cells are now primed to undergo robust follicle formation and oocyte development in follicles when re-aggregated. The percentage of surviving re-aggregated oocytes was calculated in comparison to intact transplant controls. Oocyte (green) and granulosa cell (blue) contacts and paracrine signaling factors may promote oocyte survival (+) and possibly facilitate programmed ovarian germ cell cyst break down into follicles. The cord and/or oocyte-mediated recruitment of granulosa cell clusters during ovary differentiation may also provide somatic cell signaling factors such as WNT4, R-spondin1, and Follistatin that promote oocyte survival and maturation. In addition, intact ovarian cords may facilitate the protection of oocytes from re-aggregation induced inhibitory signaling from somatic cells in the genital ridge. Thus, we define a critical window from e13.5 to e16.5 of oocyte enclosure in intact fetal ovarian cord structures for the intrinsic programming of oocytes with competence to survive and undergo further development.

Based on our results, we suggest a model in which intact ovarian cord structures that develop on e13.5 provide factors that support oocyte development (Figure 8). A notable aspect of intact ovary maturation is the development of oocytes in clusters which may facilitate cell contact and/or paracrine signal support of oocyte survival. In a previous study, intercellular bridges were not required for oocyte cyst formation and female fertility, although their absence resulted in significant peri-natal oocyte loss [14]. However, germ cell contacts have also been reported to occur by cadherin protein adhesion *in vivo *and *in vitro *as early as the PGC migratory stage and may account for oocyte cyst-like clustering and survival in the absence of intercellular bridge support [34-36]. Although re-aggregation-mediated disruption of intercellular bridges may have contributed to the reduction in oocyte numbers following transplantation, we attribute the complete absence of oocyte survival to the additional perturbation of ovarian cyst and cord structures. Furthermore, ovarian cords are comprised of pre-granulosa somatic cells, and pre-granulosa-oocyte interactions through cell contacts and/or paracrine factors may promote oocyte survival as well [37]. Cord organization of granulosa cells in close physical proximity to oocytes may also facilitate cyst break down and follicle formation [10,11,22], and the results presented here support this notion. Our results are also in agreement with a previous report of *in vitro *follicle formation from e13.5 ovaries but not from e12.5 genital ridge, confirming an important role for ovarian cord structures that develop in the intact ovary [12].

While disruption of the genital ridge resulted in an oocyte-intrinsic impairment that was independent of somatic granulosa cells, the possibility exists that other types of somatic cells in the re-aggregated genital ridge contributed to oocyte loss. Somatic endothelial and basement membrane cell types expressing PECAM and laminin were inappropriately localized in close proximity to oocytes following re-aggregation. Therefore, in addition to directly imparting oocytes with competence to survive and form follicles, intact ovarian cords that develop on e13.5 may be required to help protect oocytes from deleterious factors expressed by somatic cells in the gonad and/or mesonephros, ensuring oocyte survival and development (Figure 8). Although the mesonephros has traditionally been referenced as a facilitator of female ovary maturation and oocyte meiosis [38-40], the mesonephros also contributes to testicular cord formation and development [41,42]. In addition to the presence of meiotic oocytes [43], WNT4, R-spondin1, and Follistatin somatic cell signaling factors were required for ovary sex determination and repressed testicular endothelial and somatic cell migration from the mesonephros [44-48]. Female mice containing genetic null mutations in these factors developed disorganized ovaries with oocytes that entered meiosis but did not survive; analogous to the phenotype reported in this study. The unexplained oocyte loss in these mutant mice therefore may be an indirect consequence of ovary disorganization downstream of the mutation. In the current study, although ovarian somatic cell signaling pathways were not mutated, genital ridge disruption was sufficient to prevent re-organization of ovary structure and potential ovarian cord protection of oocytes from restrictive factors. Future studies will determine genital ridge somatic cell contribution to oocyte loss following re-aggregation and disruption of the ovarian cords.

## Conclusions

In conclusion, we report that intact ovarian cord structures that develop on e13.5 are critical for oocyte development. Thus, we emphasize the significance of intact ovary organization during mid (e13.5) to late (e16.5) gestation for programming oocytes with intrinsic competence to survive and later develop in mouse ovarian follicles after birth. Human oocytes also develop in cyst- and cord-like structures [49], and identification of genetic mutations and/or environmental toxins affecting human fetal ovary organization could provide insight into lack of human oocyte survival and development in premature ovarian failure-based cases of infertility. Additionally, the understanding of intact fetal ovarian structure and function will facilitate the development of a robust transplantation-based ovarian niche to direct the maturation of oocytes from other potential sources, such as embryonic stem cells, for the treatment of female infertility.

## Methods

### Gonad re-aggregation and kidney capsule transplantation

The transplantation system was adapted from a previous report of successful oocyte maturation following newborn ovary re-aggregation and transplantation under the kidney capsule [50]. Fetal to newborn stage female gonads with the mesonephros were dissected from wild type CD-1 mice (Charles River) and dissociated to single cell suspensions by a 10 minute 0.25% trypsinization followed by pipetting 10-20 times and re-suspension in standard media (DMEM, 10% FBS, 1% L-glutamine; Invitrogen). For e11.5 to e13.5 stages, genomic DNA was isolated from somatic tissue of each embryo and genotyped for *Sry *to confirm the gender (Zymo) [51]. The gonadal cell suspension from 4 embryos or pups was then mixed with 0.2 mg/mL of phytohemagglutinin (Sigma). Cell suspensions were pelleted at 10,000 × g for 1 minute, and pellets were incubated overnight in standard media on CM cell culture inserts (Millipore) at 37°C. Pellets, or grafts, were transplanted under the kidney capsule of bi-laterally ovariectomized CB.17 SCID recipient mice (Charles River) according to the protocol (#16146) approved by the Stanford University Administrative Panel on Laboratory Animal Care and as described in detail on the NIH Biology of Mammary Gland website link: http://mammary.nih.gov/tools/mousework/Cunha001/Pages/Written_Method.html.

### Meiotic cell spread and immunofluorescence

Following transplantation, tissues were dissociated as above and re-suspended in 20 μL of hypo-extraction buffer pH-8.2 (30 mM Tris pH 8.2, 50 mM Sucrose, 17 mM Citric Acid, 5 mM EDTA, 0.5 mM DTT, and 1% protease inhibitor cocktail (all Sigma)) for 30 minutes at room temperature. 60 μL of 100 mM Sucrose was then added, and the cell suspension was spread onto slides pre-coated with 1% Paraformaldehyde (USB Corporation) and 0.15% Triton-X100 (Sigma) in PBS pH-9.2, and dried overnight at room temperature. Slides were blocked in 4% chicken serum (Abcam) and incubated overnight at 4â—¦C with primary antibody in TBST (tris buffered saline (TBS), 1% BSA, and 0.1% Tween-20 (all Sigma)) and 1% serum. Primary antibodies included rabbit anti-SYCP3 (1:1000, Abcam, ab15092) and rabbit anti-SYCP1 (1:500, Abcam, ab15090). Slides were subsequently incubated with Alexa Fluor chicken anti-rabbit (1:1000, Invitrogen) secondary antibodies for 30 minutes, and cover slips were mounted with Prolong Gold Antifade with DAPI (Invitrogen).

### Immunofluorescence and TUNEL

Grafts were fixed overnight in 4% Paraformaldehyde (USB Corporation), embedded in paraffin, and sectioned in 5 to 8 μm intervals. In brief, sections on slides were de-paraffinized, re-hydrated, antigens unmasked by incubating in Target Retrieval Solution (Dako) at 95â—¦C for 30 minutes, permeabilized in 0.1% Triton-X100 (Sigma) for 5 minutes, blocked with 10% chicken serum in TBST overnight, and incubated with primary antibody in TBST with 1% serum for 1 hour at room temperature. Primary antibodies included anti-VASA rabbit polyclonal (1:500, Abcam, ab13840), anti-TRA98 rat monoclonal (1:500, B-Bridge, 73-003) [52,53], anti-FOXL2 goat polyclonal (1:100, Abcam, ab5096), active anti-Caspase2 (ab2251) or anti-Caspase3 (ab13847) rabbit polyclonal (both 1:100, Abcam), anti-CD31 PECAM rabbit polyclonal (1:20, Abcam, ab28364), anti-Laminin rabbit polyclonal (1:200, Abcam, ab11575), and anti-GFP rabbit monoclonal E385 (1:1000, Abcam, ab32146). After washing in TBST, slides were incubated with secondary antibody for 30 minutes at room temperature. Secondary antibodies included Alexa Fluor chicken anti-rabbit, rat, and goat (1:1000, Invitrogen), and cover slips were mounted with Prolong Gold Antifade with DAPI (Invitrogen). The terminal deoxynucleotidyl transferase dUTP nick end labeling (TUNEL) assay was performed prior to co-immunofluorescence according to the manufacturer's instructions (Roche Diagnostics).

### Fluorescence activated cell sorting (FACS)

*Oct4*-GFP gonads were dissociated to single cells with 0.25% Trypsin (Invitrogen) for 5-10 minutes at 37°C and re-suspended in standard media. Cells were strained through a 40 μm filter (BD Biosciences) and then analyzed on a BD-FACSAria cell sorting system (BD Biosciences). Post sorting, 10 to 20 thousand GFP+ germ cells were re-plated per well on a 24-well PET 0.4 μm cell culture insert (BD Falcon) for three days in standard media supplemented with a germ cell factor cocktail adapted from [33]: mouse SCF 100 ng/mL, mouse SDF1 20 ng/mL, mouse bFGF 20 ng/mL, mouse BMP4 50 ng/mL (all R&D Systems), N-acetylcysteine 1 mg/mL, Forskolin 5 μM, Retinoic Acid 1 μM (all Sigma), and CYP26 inhibitor R115866 1 μM (Johnson&Johnson). The GFP+ germ cells were then trypsinized and co-aggregated with dissociated wild type e16.5 or P2 ovarian tissue and transplanted as described above.

### Statistical analysis

Data are represented as mean +/- standard deviation (s.d.). Statistical significance was determined by unpaired Student's t-Test of unequal variance. Following transplantation, grafts from at least three separate transplants per fetal/newborn stage were sectioned and sampled by counting oocytes with nuclei in follicles on every tenth section for 150 sections by H&E stain. Follicle number per graft was normalized per section or normalized per milligram (mg) of graft tissue. For meiotic entry and progression, 100 cells from each of three separate transplants per sample were analyzed for meiotic chromosome synapsis. For oocyte survival, the numbers of TRA98+ oocytes were counted in three separate samples per time point. For apoptotic markers, at least 100 TRA98+ oocytes were assessed for co-expression of active Caspase2/3 or TUNEL.

## Authors' contributions

CRN performed study design, all experiments including transplantation and analyses, and drafted the manuscript. KMH carried out genotyping analysis as well as dissection and FACS sorting assistance, and contributed to experimental design. RARP provided financial support, conceived of the study, and participated in its design. All authors read and approved the final manuscript.
